# Antibiotic residues correlate with antibiotic resistance of *Salmonella typhimurium* isolated from edible chicken meat

**DOI:** 10.1038/s41598-025-98189-4

**Published:** 2025-04-30

**Authors:** Hala R. Ali, Esraa G. Hefny, Naglaa F. Koraney, Samah F. Ali, Mohamed I. AbdAllah, Mai A. Fadel, Sara M. Elnomrosy, Momataz A. Shahein

**Affiliations:** 1 Bacteriology Department, Animal Health Research Institute (AHRI), Agriculture Research Centre (ARC), Dokki, Giza, 12618 Egypt; 2 Food Hygiene Department, Animal Health Research Institute (AHRI), Agriculture Research Center (ARC), Dokki, Giza, 12618 Egypt; 3 Genome Research Unit, Animal Health Research Institute (AHRI), Agriculture Research Center (ARC), Nadi El-Said Street, Doki, Giza, 12618 Egypt; 4 Pharmacology and Pyrogen Unit, Department of Biochemistry, Toxicology and Feed Deficiency, Animal Health Research Institute (AHRI), Agricultural Research Center (ARC), P.O. Box.12618, Giza, Egypt; 5 Department of Virology Research, Animal Health Research Institute, Agriculture Research Center (ARC), Giza, 12618 Egypt

**Keywords:** *Salmonella*, *S. typhimurium*, Antimicrobials, Antibiotic residues, Antimicrobial resistance, Antimicrobials, Bacteria, Bacteriology

## Abstract

Irrational application and overuse of antibiotics in poultry production is associated with antibiotic residues in meat tissues which lead to the development of antibiotic resistance in pathogenic bacteria such as *Salmonella*. This study investigated the co-existence of multi-drug resistant *Salmonella typhimurium* (*S. typhimurium*) and residual traces of antibiotics in chilled chicken meat collected from Fayoum province in Egypt. Our data reported that 22.2% of chicken meat and liver were contaminated with *S. typhimurium* with 100% being drug resistant to a wide range of antimicrobials including tylosin, chloramphenicol, and oxytetracycline (OTC). The residual analysis of chicken meat samples revealed that they contained residues of 3antibacterial drugs including tylosin, chloramphenicol, OTC and its metabolite (4-epi-OTC). The statistical analysis of obtained data also revealed a relationship between *S. typhimurium* resistance and the presence of antibiotic residues in chicken samples, as isolates derived from samples with residual drug content higher than maximum residue levels (MRL) were found to be resistant to the same antibiotic. This confirms the importance of integrating sustainable practices in the veterinary field with a strict commitment to monitoring the bacterial content and residual antibiotics in foods of animal origin before marketing them, to protect the effectiveness of antibiotics for future generations.

**Supplementary Information:**

The online version contains supplementary material available at 10.1038/s41598-025-98189-4.

## Introduction

Irrational use of antimicrobials has now been identified as a major global health threat especially in developing countries where regulation of antibiotic use is not adequately considered^1–3^. One such unregulated application is the feeding of antibiotics to food-chain animals such as poultry. The benefits of antibiotic use in poultry feed and water, has been practiced and supported for many years^4–6^. However, the negative impacts of this practice, without considering the withdrawal period, may lead to the risk of antibiotic residues existing in meat tissues. The presence of these residues in food poses a risk to human health because it may be associated with allergic reaction, adverse effect on gut flora and the development of antimicrobial resistance^7^.

A number of studies have demonstrated that bacterial resistance to new antimicrobials can emerge due to selective pressure resulting from their un-regulated use, leading to increasing resistance in human and animal isolates over time. Furthermore, a direct relationship between antibiotic consumption and the emergence and dissemination of resistant bacterial strains in humans and the environment have been shown in many studies^8,9^. Consumption of antibiotic-treated livestock was highlighted as a major cause of the spread of resistant bacteria to humans^10,11^. Transmission of resistant bacteria to humans by farm animals was confirmed when high rates of antibiotic resistance were found in the intestinal flora of both farm animals and farm workers. Molecular characterization methods have recently shown that resistant bacteria and resistant genes in food chain animals reach the human body through consumption of their meat^11^.

Food chain animals may serve as important reservoirs of resistant zoonotic bacteria that can colonize the human intestinal tract and transfer resistance genes to the commensal flora. *Salmonella* is a frequent enteric pathogen in poultry, and is one of the major foodborne pathogens of zoonotic importance to public health^12^. Among *Salmonella spp*, *S. enterica* is a ubiquitous pathogen with a large host range causing various diseases^13,14^. Hosts include food chain animals or companion animals and humans, and disease symptoms range from limited gastroenteritis to invasive systemic infections with a high mortality rate^13^.

Animal-derived *Salmonella* is highly pathogenic and have been commonly implicated in outbreaks of gastroenteritis in Egyptian patients, resulting from the consumption of contaminated food^15,16^.

The presence of antibiotic residues in chicken meat and chicken products has been reported in several Egyptian cities^17,18^. Understanding the complex interplay between antibiotic use and the effects of residues in food chain animals such as chickens, in relation to the development of antibiotic resistance is critical to implementing effective approaches to mitigate this growing public health threat. Therefore, it is of utmost significance to study bacterial content of zoonotic importance and residual presence of antibacterial substances in poultry products. The current study investigated the relationship between the presence of three residual antibiotics in commercial chicken meat and antibiotic resistance of *Salmonella* isolates from the same chicken samples. This work aims to highlight the multifaceted nature of antibiotic use and emphasizes the importance of adopting sustainable practices in the poultry industry to monitor and control residual levels of antibiotics, significantly, to protect the effectiveness of antibiotics for future generations.

## Materials and methods

### Bacterial isolation and biochemical characterization

A total of 90 chilled meat samples including breast meat (30), thigh meat (30) and liver (30) were collected from commercialized chickens in 6 shops and markets of Fayoum city.

Around 10 g per sample was homogenized in buffered peptone water for pre-enrichment and incubated at 37 °C for 18–24 h. For isolation of *Salmonella*, 1 ml from the pre-enriched buffered peptone were transferred to 10 mL of selective enrichment media, Rappaport Vassiliadis Soya bean broth (RVS) and incubated further at 41 °C for 24 h. A loopful of RVS was then transferred to Salmonella Shigella agar, and further incubated at 37 °C for 24–48 h. The black colonies assumed to be *Salmonella* were then subjected for biochemical characterization using catalase and methyl red, mannitol, glucose and hydrogen sulfide production, lactose, oxidase, indole, Voges Proskauer, and urea hydrolysis tests^19^.

### Molecular characterization

The isolates were primarily tested using PCR specific for invasion protein A (*Inva*) gene^20^. Briefly, DNA extraction was performed using the QI AGEN Cat apparatus (Nos. 69504 and 69506 DNA Tissue kits) as described by the manufacturer.

Further, the extracted DNA was assayed by gene-specific PCR using Cosmo PCR Red Master Mix (Catalog No. W1020300x, Willow Fort, UK). The reaction was prepared as follow; 10 µI of COSMO PCR RED Master Mix, 2 µI of specific primers, 5 µI DNA sample and 3 µI nuclease-free water. Amplification was performed using thermal Bio-Rad thermal cycler and consisted of one cycle of initial denaturation at 95 °C for two minutes followed by 35 cycles of denaturation at 95 °C for 15 s, annealing at 52 °C for 20 s, and extension at 72 °C for 1 min; and a final extension cycle at 72 °C for ten minutes. Primers used are forward, GTG AAA TTA TCG CCA CGT TCG GGC AA and reverse TCA TCG CAC CGT CAA AGG AAC C. Size of amplified product is 284 bp^20^.

The PCR products were then separated using 1.5% agarose gel electrophoresis and ethidium bromide staining. Briefly, a combination of the PCR amplicon and gel loading buffer (50% glycerol/0.1 M EDTA, pH 8.0/1% SDS/0.1% bromophenol blue/0.0% xylene cyanole) was loaded into 1.5% agarose in 1× TBE (89 mM tris/89 mM boric acid/2 mM EDTA, pH 8.0). A 100-bp ladder was the size standard (Gibco, BRL). The gel was visualized using a gel documentation system. The size-specific DNA bands were excised and purified from gels using the QIAquick Gel Extraction Kit (Qiagen, Hilden, Germany).

The purified DNA templates were sequenced using Sanger dideoxynucleotide sequencing using the BigDye^®^ Terminator v3.1 Cycle Sequencing Kit (Thermo Fisher, USA), as instructed by the the manufacturer, utilized were reverse primers with a 3.2 p.mol. The sequencing products were purified using CentriSepTM Spin Columns (Thermo Fisher, USA), and the injection was done using capillary electrophoresis systems 3500 Genetic analyzers (Applied Biosystems, USA) sequence analysis. Multiple sequence alignments of proteins were prepared using the BioEdit Sequence Alignment Editor 7.2.5^21^. A midpoint-rooted maximum likelihood phylogenetic tree was made with Molecular Evolutionary Genetics Analysis version 5.2 (MEGA5.2 software), https://www.megasoftware.net. Then, utilizing 1000 bootstrap replicates, the tree was verified. This tree cannot operate without the matrix-based Jones–Taylor Thornton (JTT) model^22^. Phylogenetic trees were inferred using the Maximum Likelihood method built into the Molecular Evolutionary Genetics Analysis version 11.0.13 (MEGA11) software (https://www.megasoftware.net). and bootstrapping more than a thousand replicates were used to estimate the topology^22^.

### Serotyping of *S. enterica subsp.* Isolates

The traditional method to determine a Salmonella serotype is a phenotypic method^23^, based on the White–Kauffmann–Le Minor scheme (Grimont and Weill, 2007)^24^. The serotype is determined by agglutination of the bacteria with specific antisera to identify variants of somatic (O) and flagella (H) antigens (Lillidale Diagnostics Co., UK).

### Antibiotic sensitivity profiling

Isolates of *S. typhimurium* were examined against several antibiotic discs (Sigma, Cairo) according to the Clinical and Laboratory Standards Institute (CLSI) standard 2021^25^. Briefly, purified colonies were cultured on Muller Hinton broth overnight, the inoculum optical density was then adjusted to 0.5 MacFarland Standard. 100 µl was spread out on the Muller Hinton agar, and the surface of the inoculated plates was loaded with antibiotic discs of gentamycin (CN) (10 µg), tylosin (TY30 µg), levofloxacin, ciprofloxacin (CIP 5 µg), colistin (CL10 µg), Cefotaxime (30 µg), amikacin (AK 5 µg), amoxicillin (AM10 µg), and imipenem (IPM 10 µg), chloramphenicol (C30µg), and oxytetracycline (TE 30 µg). Following incubation for 24 h at 37 °C, each antibiotic’s inhibition zones were determined and recorded.

### Antibiotic residual analysis

#### Seven plate bioassay technique

The test depended on the inhibition zone of different antimicrobials against seeded plates with sensitive bacteria^26^. Tissue preparation for the seven plate bioassay was performed by mixing 10 g of diced tissue sample with 40 ml of appropriate buffer for each microbial residue. The sample was mixed well for 1 min and incubated for 45 min before injecting 200 µl of the extract into wells of each seven plates. Plate 1: Using *Bacillus cereus* and penicillinase at pH 4.5, plate 1 contained tetracycline residue. Plate 2: Beta-lactam residues were detected on Plate 2 by *K. rhizophila* at pH 6.Plate 3: prepared for confirming penicillin-free at pH 6 for *K.rhizophila*. Plate4: *B.subtilis* utilized in Plate 4 to look for streptomycin or dihydrostreptomycin residues and penicillinase at pH 8. Plate5: measured the residues of erythromycin and tylosin by *K. rhizophila* ATCC 9341 at pH 8 and penicillinase. Plate 6: B. subtilis spore suspension used for Plate 6 with penicillinase at pH 7.2 for sulphonamide residues. Plate 7: At pH 8, aminoglycoside residues containing *S. epidermidis* and penicillinase were detected. In order to interpret the results, each plate in the seven-plate method had its inhibition zone diameter for the unknown and a standard reference antibiotic (penicillin, in plate 3’s case). The results were computed using the pre-prepared standard curve. For the positive samples, the inhibition zone’s diameter shouldn’t be less than 1–2 cm.

#### High-performance liquid chromatography (HPLC) analysis

HPLC was used to confirm the positive tissues samples microbiologically which detected tylosin^27^ and oxytetracycline (OTC)^28^ Chloramphenicol was tested against a chloramphenicol disc and confirmed its residues by HPLC^29^.

Tylosin was extracted from tissue by adding 10 ml of chloroform and 1 ml of phosphate buffer (Na_2_ HPO and KH_2_ PO4, 0.07 M, pH 8.5) to 5 g of muscle sample. This was followed by shaking and centrifugation for 20 min at 1932 g. The collected organic phase was dried at 400 °C and reconstituted with 4 ml of acetonitrile: H_2_O (8:2) and dichloromethane. Another shaking and centrifugation were applied to collect the organic phase. The extraction step was then repeated with dichloromethane followed by drying of all the collected organic phase and re-dissolving with 200 µM mobile phase (acetonitrile: 0.04 M Na_2_ HPO_4_) (33:66 v/v). Then 50 µl of sample was injected into the HPLC system.

OTC and its metabolite were extracted for HPLC by mixing 2 g of homogenized chicken sample with 0.1 g of citric acid, 1 ml of nitric acid (30%), 4 ml of methanol, and 1 ml of deionized water. Samples were then vortexed and ultrasonicated for 15 min followed by centrifugation for 10 min at 5300 rpm. The samples were then filtrated using a 0.45 μm nylon filter and 20 µl of each filtrate was injected into HPLC separation by the mobile phase consisted of acidified water with H_2_SO_4_ pH 2.1 to acetonitrile (85:15 v: v).

To detect chloramphenicol residue, 10 g of the chicken sample was homogenized in 40 ml of distilled water and filtered followed by cleaning up with a C18 cartridge which was then eluted with 50 ml of dichloromethane. Then, the organic part was evaporated at 40 °C and the residue was dissolved with 15 ml of dichloromethane, 300 µl of distilled water and 2 ml of toluene. Samples were mixed and centrifuged. The organic phase was then discarded and the extraction step was repeated using 1.5 ml of fresh toluene. The aqueous phase was filtered to be injected for HPLC column by 20 µl to be separated by methanol/H_2_O /acetic acid 45/55/0.1.

Multiwave detector (UV) HPLC detector (AGILENT, Japan) with quaternary pumps were used for HPLC quantification. A 5 μm Kromasil C18 ODS column (250 mm × 4.6 mm id) from the Netherlands was used for the separation process, which involved the use of reverse-phase (RP) technology at room temperature. Data analysis and method control were performed using Agilent Open Lab Chemstation LC B.04.03 Software (https://innovtrendlab.com/product/agilent-chemstation-lc-b-04-03-software/).

Standards for HPLC analysis (tylosin, OTC with its main metabolite 4-epi-OTC, chloramphenicol, and HPLC grade solvents) were obtained from Sigma Aldrich (Merck, Darmstadt, Germany). All analytical solvents were purchased from commercial sources. Deionized water was purified by Milli-Q system (Millipore, Bedford, MA).

#### Verification of the analytical HPLC methods

Tylosin, OTC, and, chloramphenicol analytical HPLC methods were verified according to the international conference on harmonization guidelines of technical requirements for registration of pharmaceuticals for human use^30,31^.The methods were checked for accuracy, repeatability, specificity (retention time), linearity and range.

Statistical analysis of the data was performed using SPSS version 22 via Chi-Square and Fisher’s Exact tests. The Chi-Square test was used to determine the association between chicken samples positive for antibiotic residues and *S. Typhimurium isolates* resistant to the same antibiotics. All statistical tests were performed using P value < 0.05.

## Results

### Isolation and identification of *Salmonella enterica*

**Fig. 1:**
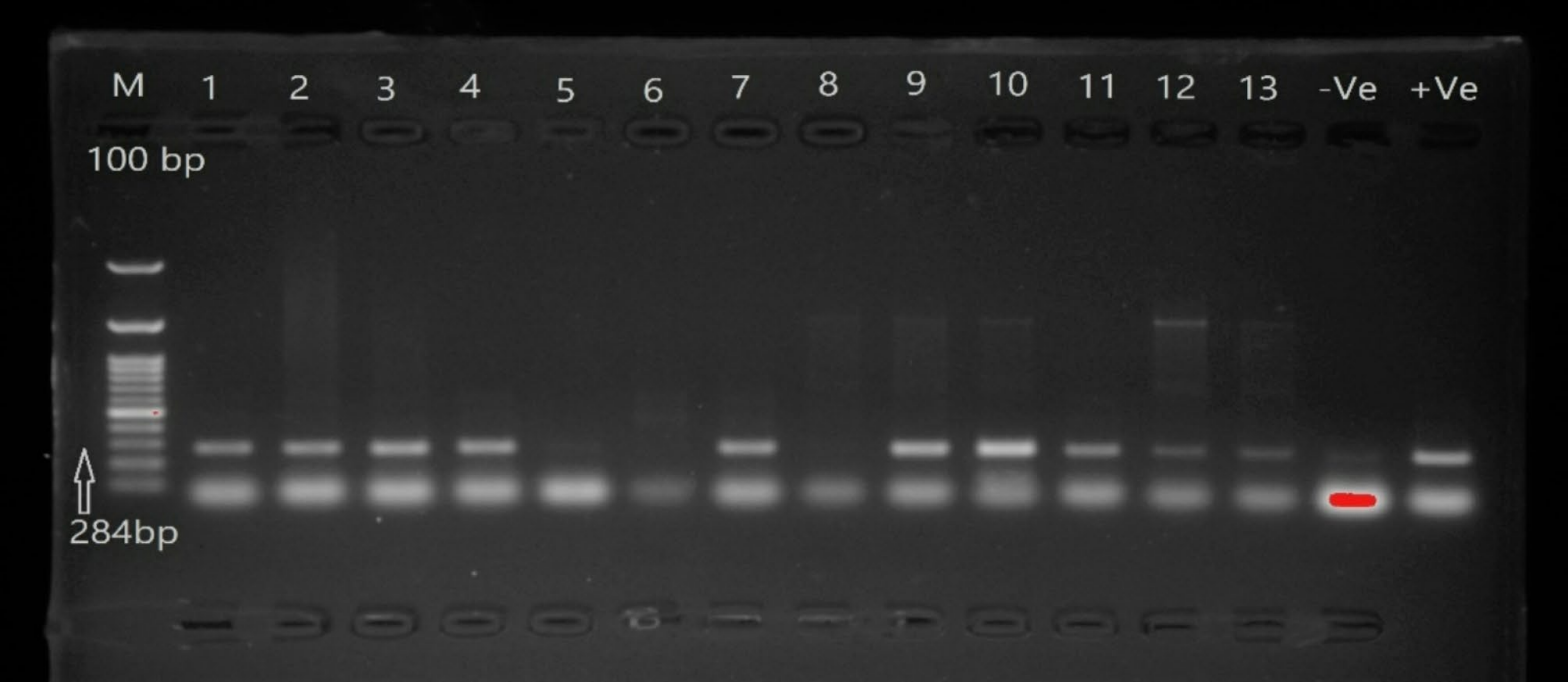
The PCR amplicon of *Inva* gene in *Salmonella* isolates with positive band at 284 bp. Lane M: is 100 bp ladder, Lane 1–4, 7 and10–13: positive, Lane 5, 6 and 8: negative, −Ve: negative control and + Ve control.

**Fig. 2:**
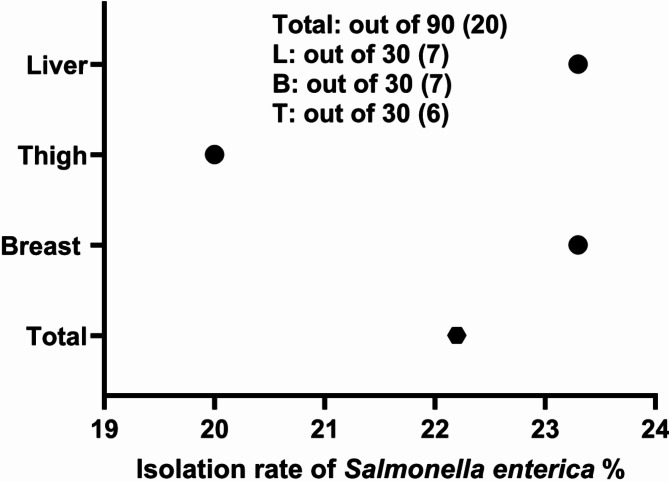
The prevalence rate of *S. enterica* serovar *typhimurium* among the different tested chicken parts.

**Fig. 3:**
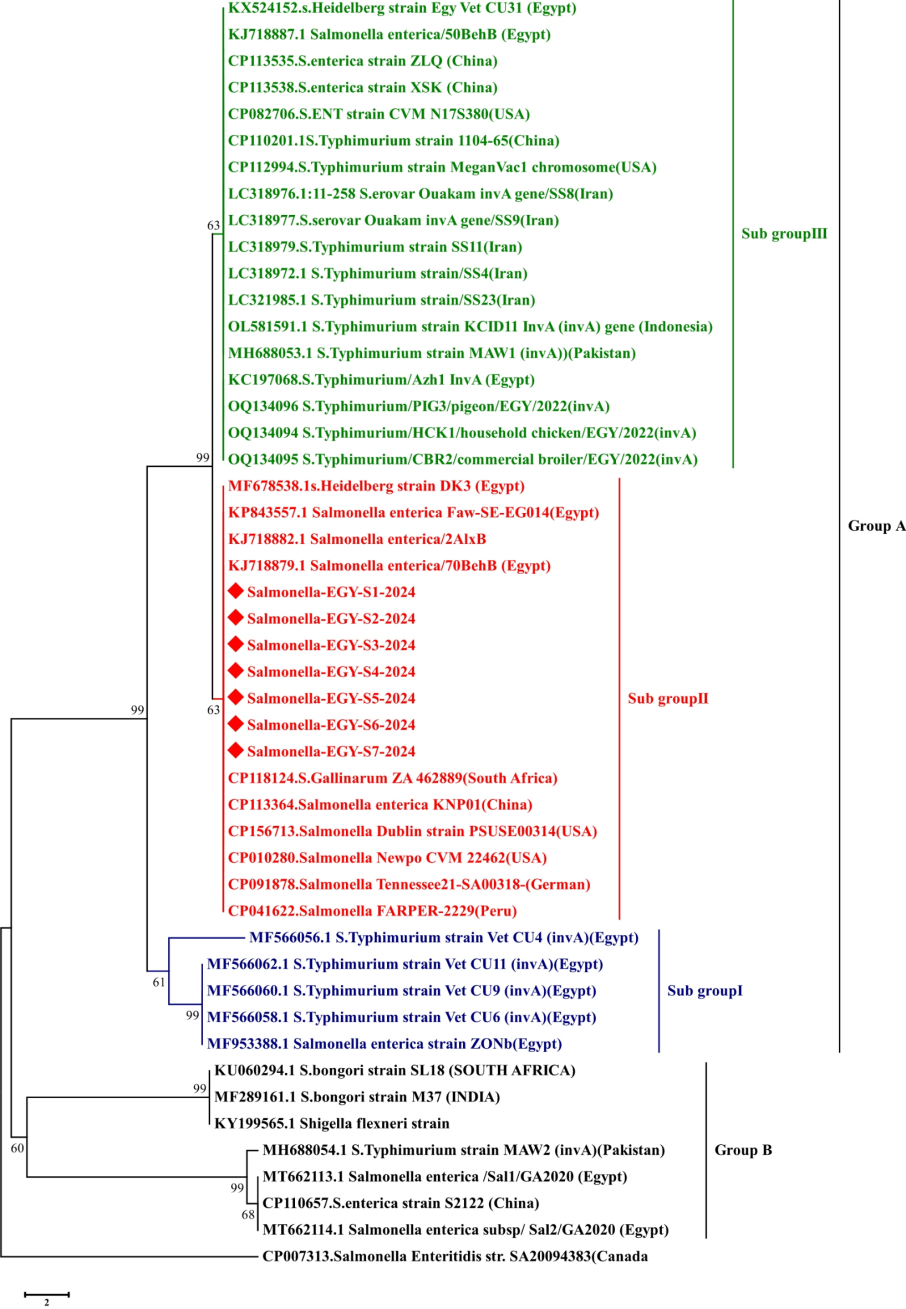
Phylogenetic analysis of invasion protein (*inva*) gene, of *S. enterica subsp*. The partial nucleotide sequences from different strains of *S. enterica* subsp., were obtained via NCBI Resource. The phylogenetic analysis was performed using MEGA6. Construction with the maximum-likelihood (ML) analysis of evolutionary distances determined by the GTR + G + I model. NJ and ML bootstrap (×1000) Consensus neighbour-joining trees were obtained from 1000 bootstrap replicates. The red-rhombus indicates strains under study.

**Fig. 4:**
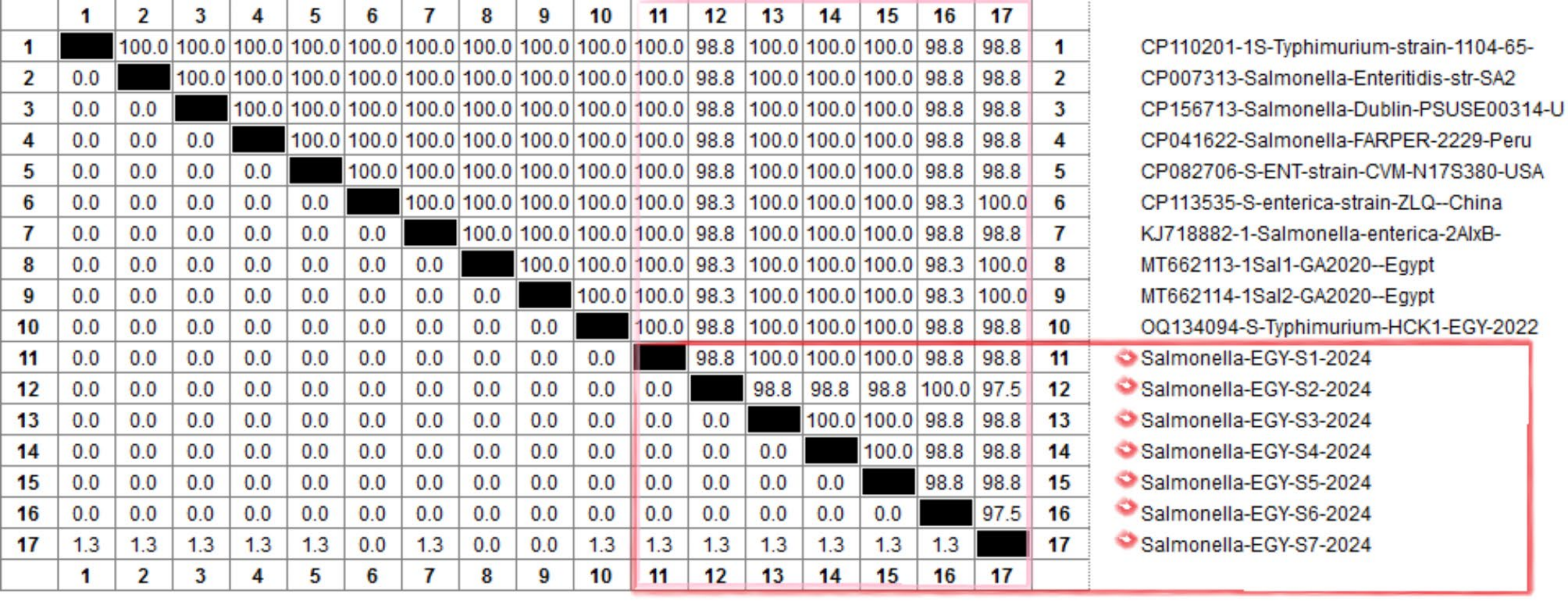
Nucleotide identities of partial *Inva* gene, compared to other selected Egyptian strains, field, and strains available on GenBank.

Out of 90 samples of chicken meat parts, 23 isolates were suspected to be *Salmonella spp.* by bacteriological analysis, showing colourless colonies with a black center on SS agar media and colourless colonies on MacConkey agar. The bacteriological positive colonies were biochemically confirmed as positive for catalase and methyl red, mannitol, glucose and hydrogen sulfide production, while negative for lactose, oxidase, indole production, Voges Proskauer, and urea hydrolysi. As shown in Fig. 1, seven isolates from breast meat, six isolates from thigh meat, and seven isolates from liver were positive for the *Inva* gene at 284 bp and in total, 20 isolates (22.2%) were further confirmed as *S. enterica subsp.* by partial sequencing of the *Inva* gene (Fig. 2). The phylogenetic tree analysis showed that the Egyptian strains were clustered with *S. enterica subsp.*, which grouped into two major groups (gr A and gr B). Group A subdivided into 3 sub-groups, and the strains under study were clustered within subgroup 2 along with some Egyptian strains and, USA, and German strains (Fig. 3). As presented in Fig. 4, the amino acid identity analysis revealed that the study isolates had a high identity percentage with each other, ranging from 97.5–100%.and show 100% similarity to recently published Egyptian strain in 2020. Further, the slide agglutination tests using O and H antisera confirmed that *S. enterica subsp. enterica* belong to the serovar *Typhimurium*.

**Table 1:** Antibiotic profile of *S. typhimurium* isolated from chicken meat (Breast (B), thigh (T) and liver (L)).

Antibiotic group	Antibiotic	Salmonella enterica isolates			
		*n*^L^ (7)	*n*^B^(6)	*n*^T^(7)	Total *N*: 20 (100%)
Tetracycline	Oxytetracycline	7	6	7	20 (100%)
Amphenicol	Chloramphenicol	7	6	7	20 (100%)
Macrolides	Tylosin	7	6	7	20 (100%)
Aminoglycosides	Amikacin	2	-	2	4 (20%)
	Gentamycin	6	6	6	18 (90%)
Quinolones	Levofloxacin	6	5	6	16 (85%)
	Ciprofloxacin	7	5	5	16 (85%)
Third-generation cephalosporins	Cefotaxime	2	1	2	5 (25%)

Furthermore, *S. typhimurium* isolates were subjected to antibiotic analysis using the disk diffusion method against 8 antimicrobials. The results (Table 1) showed that 100% of the isolates were resistant to tylosin, chloramphenicol, and oxytetracycline, while 90% were found resistant to gentamicin. 85% of the isolates showed resistant to both levofloxacin and ciprofloxacin. Resistance to amikacin and cefotaxime was detected in 25% and 20% of the isolate, respectively.

### Antibiotic residues

**Fig. 5:**
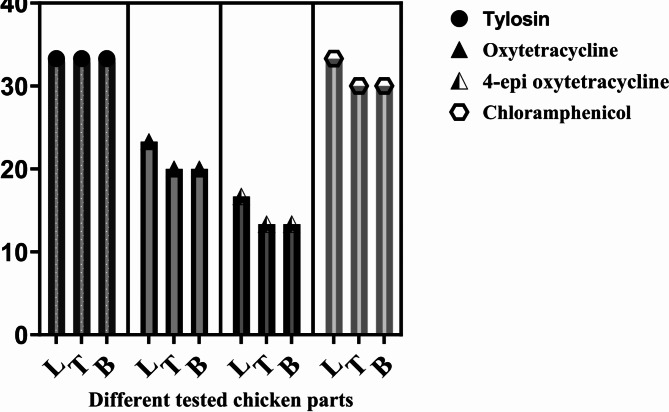
The incidence of antibiotics residues among the analyzed chicken parts (L: Liver, B: Breast & T: Thigh).

**Fig. 6:**
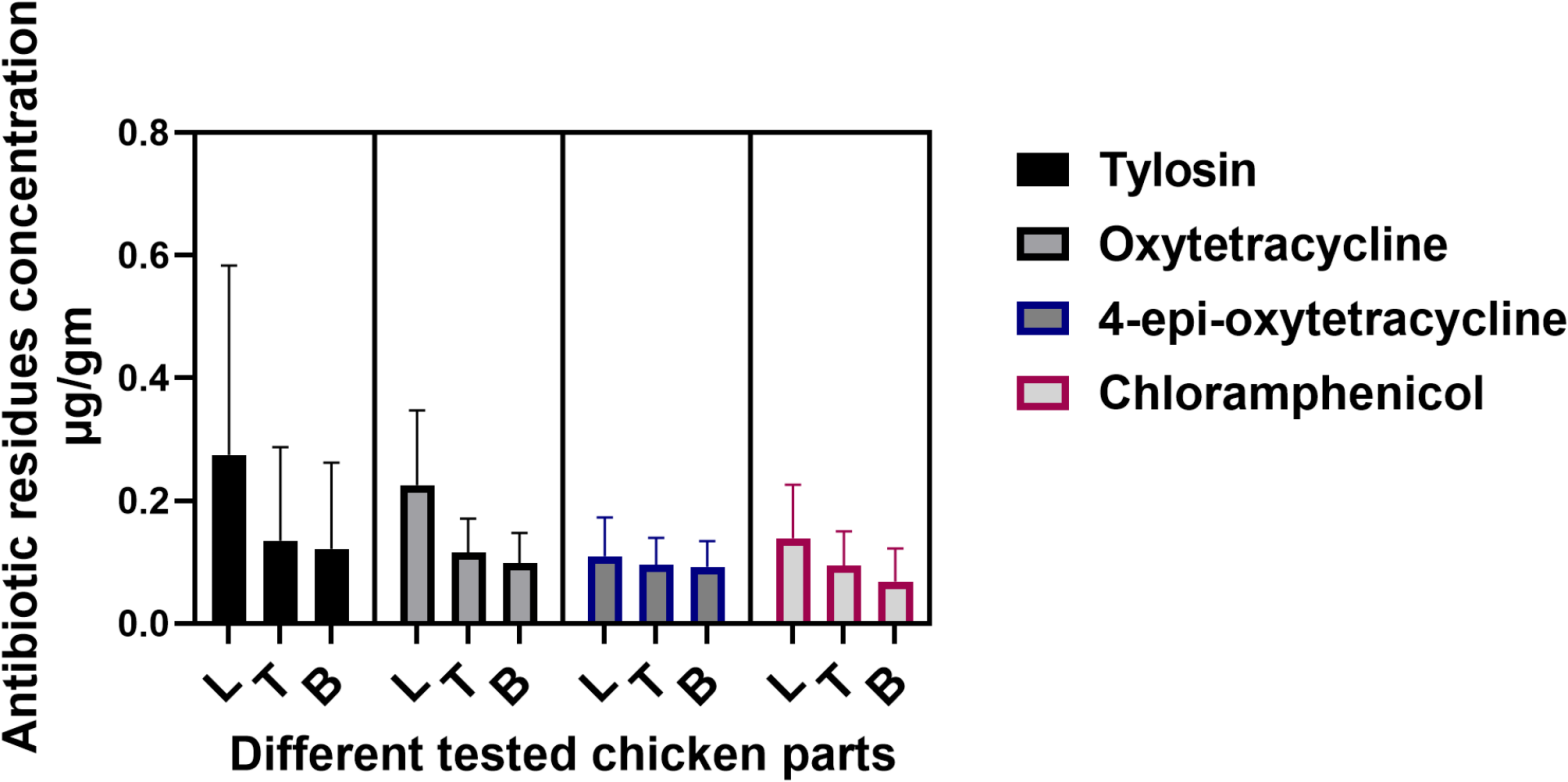
Average concentrations of antibiotic residues in tested chicken samples (L: Liver, B: Breast & T: Thigh) from Fayoum.

Antibiotics residues concentrations were determined by verified RP-HPLC methods as illustrated in Table S1. As shown in Fig. 5, antibiotic residues including tylosin, chloramphenicol, OTC and its metabolite epi4-OTC were detected in the chicken samples tested. Tylosin residues were the highest in all chicken parts tested (33.3%), followed by chloramphenicol which recorded 33.3, 30 and 30% in liver, thigh and breast, respectively. OTC was identified in 23, 20, and 20% of the liver, thigh, and breast muscles, respectively, while its metabolite was present in a smaller percentage of samples, ranging from 13.33% in the thigh or breast muscle to 16.67% in the liver samples. The result presented in Fig. 6, indicated that liver was associated with the highest incidence and concentrations of antibiotic residues followed by thigh and breast muscles. The tylosin residue concentration was ranked first in all chicken parts tested with average concentration 0.27, 0.135 and 0.121 µg/g in liver, thigh and breast muscle respectively, followed by OTC (0.225, 0.116, 0.098 µg/gm in liver, thigh and breast muscle respectively). While the average concentrations of 4-epi-OTC, and chloramphenicol were very similar, ranging from 0.109 to 0.139 µg/g in liver, 0.094 to 0.095 µg/g in thigh muscles, and 0.0682 to 0.0922 µg/g in breast muscle.

Based on the MRLs as detected by European union^32^, the residual level of chloramphenicol was above the MRL in 33.3% and 30% of liver and thigh or breast muscles respectively, while the level of oxytetracycline and its metabolites exceeded the MRL in 10% each of liver and both breast and thigh muscle samples. The tylosin residue level also exceeds the MRL in 20% of the liver sample and 10% of the breast or thigh muscles (Table S1).

As presented in Table 2, out of 12 chicken samples containing tylosin residues above the MRL, 7 samples tested positive for tylosin-resistant *S. typhimurium*. Also, 9 chicken samples containing total OTC and epi-OTC residues above the MRLs, as well as chloramphenicol residue, were tested positive for oxytetracycline/chloramphenicol-resistant *S. typhimurium*. However, 6 samples and 19 samples tested positive for OTC and chloramphenicol residues above MRL respectively, were bacteriologically negative for *S. typhimurium*. On the other hand, 4 out of 36 chicken samples that were free of the tested antibiotics residues, were tested positive for resistant *S. typhimurium.*

**Table 2:** The co-existence of antibiotic residues and resistant *Salmonella typhimurium* isolates in chicken samples (L: liver, B: breast & T: Thigh).

Detected antibiotics above MRL	Number of samples	Permissible limit* (µg/g)		Average residues concentration (µg/g)			*S. typhimurium*	Phenotypic resistance	P value
		Liver	Muscle	L	T	B			
Tylosin (n: 12)	7	0.1	0.1	0.354	0.163	0.153	+	Tylosin	0.000*
	5			0.159		0.4	−	−	
OTC & 4-epi-OTC (n:15)	9	0.3	0.1	0.406	0.132	0.14	+	OTC	0.001*
	6			0.23	0.203	0.191	−	−	
Chloramphenicol (n: 28)	9	−	−	0.128	0.07	0.06	+	Chloramphenicol	0.139
	19			0.128	0.07	0.04	−	−	
No antibiotics (n: 36)	4			−	−	−	+	Tylosin, OTC & chloramphenicol	0.034*
	32			−	−	−	−	−	

*Statistical significant relationship at P value < 0.05.

## Discussion

The widespread use of antibiotics in poultry farms either for therapeutic purposes or to promote growth have been recognized as a major threat to public health^1,2^. The negative consequences of antibiotic abuse include changes in the resistance patterns of bacteria susceptible to antibiotics and selection of resistant bacteria that could be transferred to humans through the food chain. Chicken is the most popular and consumed food around the world. To meet the constant demand for this cheap source of protein, antibiotics are irrationally used in broiler farms^33^. This practice without proper regulation results in residues of antibiotic compounds appearing in edible chicken tissues. Traces of antibiotic substances in chicken tissues, even below the permissible limit, are one of the possible causes of antibiotic resistance in human and animal pathogens^7^.

*Salmonella* is one of the foodborne pathogens commonly found in poultry, and its resistance to antibiotics poses major challenges for the treatment and prevention of infections in humans^12^. Thus, the current study was to determine whether there is a possible correlation between the presence of antibiotic residues in chicken samples and the antibacterial resistance of *Salmonella* isolates derived from the same chicken samples.

To probe this, 90 chicken samples were randomly collected from different shops located in Fayoum, one of the Egyptian governorates, which has a large poultry industry. The classical bacteriological and biochemical analysis revealed that 22.2% (20/30) of the different chicken parts were positive for *Salmonella* with the prevalence of positive samples in liver, breast, and thigh muscle reaching 23.3%, 23.3%, and 20%, respectively (Fig. 2). The bacteriologically and biochemically positive *Salmonella* isolates were found positive for *Inva* gene using conventional PCR approach. The phylogenetic tree as well as sequence analysis of *Inva* gene reported significant similarities to those in *S. enterica subsp*. strains in Egypt and other countries. *S. enterica* is resilient and adaptable to different environments, making them more likely to survive and proliferate in poultry farms. 50% of the 2500 *Salmonella* serotypes, identified to date, belong to *Salmonella enterica subsp. enterica*, which accounts for the majority of salmonellosis cases in humans^12^.

The *S. enterica* isolates were further confirmed by serotyping using the agglutination tests and the isolates were determined to belong to serovar *typhimurium*.

Compared to our results in Fayoum, a greater number of chicken carcasses 29.4% (50/170) were found positive for *S. enterica* in Sharkia Governorate^34^ while a smaller percentage (8.3%) of positive raw chicken were detected in the Nile Delta, Egypt^35^. On the contrary, there was a noticeable increase in the isolation rate of *S. enterica* (39%) from retail chicken carcasses in Mansoura Governorate^36^. Another study discovered *Salmonella spp.* in 10.9% of chicken samples collected from four Egyptian governorates: Dakahlia, Kafr El-Sheikh, Damietta, and Port Said during 2017^37^.

Based on our result and previous studies in Egypt, there is a significant variation in the incidence of *Salmonella* in poultry farms between the different Egyptian cities. This might be due to a range of factors including differences in farm management practices such as the biosecurity measures and the quality of feed and water. In addition to environmental factors, and regulatory standards which impacts the survival and proliferation of bacteria in poultry farms. Similarly, regional variations in the *Salmonella* prevalence among poultry farms of the US and UK have been attributed to environmental conditions and biosecurity measures^38^,^39^.

The antibiotic profiles of all isolates (Table 1) were established against a range of antibiotics commonly used in broiler farms. All twenty isolates were found to be resistant to three antibiotics: tylosin, oxytetracycline, and chloramphenicol, which belong to the macrolides, tetracyclines, and amphenicols, respectively. This is consistent with previous reports indicating that *Salmonella* isolates in chickens were often resistant to antibiotics commonly used in poultry farms such as tetracycline and chloramphenicol^40^. Our result also showed 85% resistant to two quinolone antibiotics (levofloxacin and ciprofloxacin), while 25% and 20% of the isolates showed resistance to one of the cephalsporines (cefotaxime) and amikacin, respectively. Consistently, the veterinary inspection in the USA reported a remarkable increase in resistant *Salmonella* isolates to ciprofloxacin and cefotaxime, recovered from chicken sources^41^. Despite the fact that quinolones and cephalosporin are critically important drugs used in treating severe human infection, farmers and breeders still use these antibiotics in livestock. This misuse promotes the selection and proliferation of resistant strains in animals, humans and in the environment. This data indicated that all the *S. typhimurium* isolates were resistant to a broad spectrum of antibiotics from six antimicrobial classes which identify them as extensive drug-resistant isolates. A review study by *Saraiva et al.*., reported an increasing frequency of multi drug resistant *Salmonella* isolates from chicken every year all over the world^42^. The potential incorporation of clinically relevant bacteria in food such as resistant *Salmonella* strains represents a critical issue at the intersection of public health and food safety. This might narrow antimicrobial treatment options and compromise their efficacy in cases of infection.

Further, chicken meat samples were specifically tested for the presence of chloramphenicol, tylosin, and oxytetracycline due to their significant health risks, and widespread use in broilers farms. Chloramphenicol is particularly targeted because of its potential to cause serious toxicity such as aplastic anemia in humans, a condition that can be fatal in susceptible people when exposed to even a single molecule, and it is therefore banned from use in farm animals in several countries^43^. Although tylosin and oxytetracycline are not banned, they were tested due to their significant role in promoting antibiotic resistance, which in turn may impact human health. These antibiotics were mainly selected for testing based on their tendency to leave residues in meat tissues thus increasing the risk of adverse health effects from consuming residue containing meat. This is why producers must comply with food safety regulations designed to protect consumers from harmful levels of antibiotics in food chain animals.

To detect and quantify the antibiotic residues in the examined chicken samples, they were primarily tested using seven plate bio-assay techniques followed by confirmation of the antibiotics existence and concentration using the HPLC. The residual analysis detected three antibiotic compounds including tylosin, chloramphenicol, and OTC with its metabolites (epi4-OTC) in chicken liver, thigh and breast muscles at variable percentage and concentrations as shown in Figs. 4 and 5. The highest incidence and concentration of the tested antibiotics were reported in liver samples followed by thigh then breast muscles. In line with a review study targeted a number of published works from Arab countries, to conclude that the concentration and presence of antibiotic residues were highest in liver compared to other edible chicken tissues^44^. This is mainly due to the major role the liver plays in filtering and metabolizing any toxins or chemical compounds, and often stores residues until they are processed and eliminated. El-Tahir et al. also found that the residual concentration of several antibiotics, including oxytetracycline, tylosin, sulfamethazine, gentamicin, and enrofloxacin, was lowest in chicken breast meat^45^.

Among the antibiotic residues discovered, the detection rate of tylosin residue was ranked first in the three types of chicken parts but exceeds the allowable limit in only 20% and 10% of liver and meat samples respectively. Chloramphenicol residues % was ranked second in all chicken parts with concentration above MRL, followed by oxytetracycline and its metabolite, exceeding the MRL in 10% of each chicken part. Conversely, Hakem et al., reported % of tetracycline residues were the highest in fresh and frozen chicken samples, followed by macrolides and then aminoglycosides^46^. A higher percentage (80%) of chicken samples investigated in Pakistan, were found containing tylosin, oxytetracycline, amoxicillin and enrofloxacin above MRL^47^.

Tylosin is one of the macrolides that is widely used as a feed additive in livestock globally. The United States was the first country to develop and approve its use in livestock, especially chickens and pigs, to enhance weight gain^48^. Macrolides are critically important antimicrobials in human health and used to treat some foodborne bacterial diseases, such as *Salmonella* and *Campylobacter jejuni*. Resistance to tylosin can also confer cross-resistance to other macrolides, such as erythromycin and azithromycin, due to similar mechanisms of action and target sites. A systematic review and meta-analysis study on beef cattle found that tylosin increases the proportion of macrolide-resistant enterococci in the cattle gastrointestinal tract^49^. Oxytetracycline and chloramphenicol are also commonly used in poultry as growth stimulants. This preference is demonstrated by the registered sales of these drugs, which can reach 6149.9 kg for amphenicol and 1631.9 kg for tetracycline, most of which are intended to be taken orally^50^. Therefore, the presence of residues of these compounds in chicken meat may be attributed to lack of monitoring the withdrawal period before marketing previously antibiotic treated chickens. The presence of chloramphenicol residues in meat tissues could be a sign of illegal use by farmers along with inadequate monitoring of food safety measures that allow banned antibiotic compounds to be used.

Our data highlights the importance of ensuring adherence to strict guidelines and creating a culture of transparency and responsibility in the food production chain to protect consumer health. Governments and food safety authorities must ensure compliance through proper testing and penalties for violations especially regarding the use of banned substances, in parallel with educating farmers and veterinarians to avoid misuse of antibiotics.

Both residual and bacteriological analysis demonstrated that 7 chicken samples contained tylosin residues and tylosin-resistant *S. typhimurium*. Also OTC and chloramphenicol residues appeared in 9 chicken samples that tested positive for resistant *S. typhimurium* isolates to both antibiotics (Table 2).

A highly significant association was detected between the presence of tylosin residues above MRL and isolation of tylosin resistant- *S. typhimurium* (P value = 0.001) from the same chicken samples. OTC residues above MRL were also significantly related to the isolation of OTC- resistant strains of *S. typhimurium* from the same chicken samples (P value < 0.05). On the contrary, no significant relationship was found between the presence of chloramphenicol residues and chloramphenicol-resistant *S. typhimurium* in the same chicken samples. In addition, the absence of residue of the three tested compounds was found to be significantly associated with negative isolation of *S. typhimurium* (P value < 0.05).

These data refer to a potential effect of antibiotic residues on the development of resistant *S. typhimurium* strains. This resistance can occur due to selective pressure resulting from exposure to antibiotics, leading to the persistence and spread of resistant bacteria among poultry populations.

The co-existence of antibiotic residues and resistant *S. typhimurium* strains in chicken samples indicate that the resistance developed in the isolates is due to exposure of bird flora to these antibiotics. However, four isolates showed resistance to tylosin and chloramphenicol and were recovered from residue-free samples of the three antibiotics tested. This might indicate a cross contamination in the slaughter houses or preparation, packaging or retail premises.

Despite this, the study provides some evidence of the potential effect antibiotic residues might have on driving *Salmonella* resistance in poultry further study including statistics on wide range of samples is required.

Several studies have proposed alternatives to antibiotics, such as natural feed supplements, to promote weight gain in broilers while addressing concerns regarding antibiotic resistance. These natural supplements including probiotics or plant-based feed additives have shown potential not only in promoting better growth performance but also in reducing the incidence of *Salmonella* infections and the shedding of *Salmonella* by infected birds. These studies aim to improve poultry health and productivity while minimizing the risks associated with antibiotic use in food chain-animals^51–52^.

Furthermore, this study recommends the implementation of a control program with effective measures to reduce and eliminate *Salmonella* in poultry farms such as the European Union eradication program that includes monitoring, testing and reporting^53^. In addition, vaccination and risk-based approaches are being followed that focus efforts on high-risk areas to improve the effectiveness of control measures.

## Conclusion

Excessive use of antibiotics in poultry farms greatly contributes to residues appearing in edible meat tissues. This also promotes the selection and proliferation of resistant strains particularly potential food-borne pathogens such as *Salmonella*. This study detected co-existence of antibiotic residues in broiler meat samples along with *S. typhimurium.* strains resistant to same antibiotic compounds. This data infer a possible relationship between feeding antibiotics to broiler, residues in their tissues, and selection of *S. typhimurum* resistance to antibiotics commonly used in broiler farms. Our data confirms the importance of adopting sustainable practices in the veterinary field and strict adherence to monitoring the bacterial content and residual antibiotics in foods of animal origin before marketing them, to protect the effectiveness of antibiotics for future generations.

## Electronic supplementary material

Below is the link to the electronic supplementary material.

Supplementary Material 1

## Data Availability

The data generated and analyzed in the current study has been deposited in the GenBank under the following accession numbers: BankIt2903852Salmonella-EGY-S1-2024 PQ741799BankIt2903852Salmonella-EGY-S2-2024 PQ741800BankIt2903852Salmonella-EGY-S3-2024 PQ741801BankIt2903852Salmonella-EGY-S4-2024 PQ741802BankIt2903852Salmonella-EGY-S5-2024PQ741803BankIt2903852Salmonella-EGY-S6-2024 PQ741804BankIt2903852Salmonella-EGY-S7-2024PQ741805.
