# Supplementary material for: Antibiotic residues correlate with antibiotic resistance of Salmonella typhimurium isolated from edible chicken meat
Source: Sci Rep. 2025 Apr 30;15:15165. doi: 10.1038/s41598-025-98189-4 (PMC12043935; doi:10.1038/s41598-025-98189-4)
Supplement: Supplementary file 1 — Supplementary Material 1 [file 41598_2025_98189_MOESM1_ESM.docx]

**Antibiotic residues correlate with antibiotic resistance of *Salmonella Typhimurium* isolated from edible chicken meat**

**Hala R Ali^1^*, Esraa G Hefny2**, **Naglaa F Koraney1, Samah F. Ali^1,^ Mohamed I AbdAllah^3^**, **Mai A. Fadel^4^, Sara M Elnomrosy^3^, and Momataz A. Shahein^5^**

1. Bacteriology Department, Animal Health Research Institute (AHRI), Agriculture Research Centre (ARC), 12618 Dokki, Giza, Egypt.
2. Food Hygiene Department, Animal Health Research Institute (AHRI), Agriculture Research Center (ARC), 12618 Dokki, Giza, Egypt
3. Genome Research Unit, Animal Health Research Institute (AHRI), Agriculture Research Center (ARC), Nadi El-Said Street, Doki, Giza 12618, Egypt.
4. Pharmacology and pyrogen unit, Department of Biochemistry, Toxicology and Feed Deficiency, Animal Health Research Institute (AHRI), Agricultural Research Center (ARC), Giza, P.O. Box.12618, Egypt.
5. Department of Virology Research, Animal Health Research Institute, Agriculture Research Center (ARC), Giza 12618, Egypt.

*Corresponding author: Hala R. Ali,

Email: [alihala312@gmail.com](mailto:alihala312@gmail.com)

**Table (S1): HPLC-verification parameters of the results.**

| **Parameters** | **Tylosin** | **OTC** | **4 epi OTC** | **Chloramphenicol** | **Acceptance criteria** |
| --- | --- | --- | --- | --- | --- |
| **Range** | **0.05-2µg/g** | | | | At least 6 expected standards |
| **Regression equation** | y=2.959x - 150.89 | y = 0.2213x + 0.014 | y = 0.2002x + 0.1948 | y = 2.8001x - 0.1059 | Assed the calibration curves of calculated standards |
| **R^2^** | 0.998 | 1 | 0.9999 | 0.9998 | At least 0.99 |
| **Repeatability** | 0.78 | 0.93 | 0.8 | 0.68 | CV%≤1 |
| **Accuracy** | 93.1±1.1 | 98.2±0.9 | 95.3±1.2 | 89.2±0.8 | RSD should be ≤2% |
| **Retention Time (RT)min.** | 2.1±0.03 | 4.2±0.07 | 5.1±0.1 | 10.3±0.01 | Specify the tested analyzed without impurities |

*CV: Coefficient of variance; RSD: relative standard deviation.

**Table (S2): Calculated % of antibiotics that exceeded recommended MRLs in broilers tissues.**

| **Antibiotics** | | **% of unaccepted samples (exceed MRLs)** |
| --- | --- | --- |
| **Tylosin** | **Liver** | 20% |
|  | **Muscles** | 10%: Thigh  10%: Breast |
| **Sum of OTC & 4- epi -OTC** | **Liver** | 10% |
|  | **Muscles** | 10%: Thigh  10%: Breast |
| **Chloramphenicol** | **Liver** | 33.3% |
|  | **Muscles** | 30% |
